# Remote family education and support program for parents of patients with adolescent and early adulthood eating disorders based on interpersonal psychotherapy: study protocol for a pilot randomized controlled trial

**DOI:** 10.1186/s40337-024-01013-z

**Published:** 2024-05-17

**Authors:** Fujika Katsuki, Norio Watanabe, Masaki Kondo, Hanayo Sawada, Atsurou Yamada

**Affiliations:** 1 Department of Psychiatric and Mental Health Nursing, Nagoya City University Graduate School of Nursing, 1 Kawasumi, Mizuho-cho, Mizuho-ku, Nagoya, Japan; 2 Department of Psychiatry, Soseikai General Hospital, 101 Shimotoba, Hiroosa-machi, Fushimiku, Kyoto, Japan; 3 National Center for Cognitive Behavior Therapy and Research, National Center of Neurology and Psychiatry, 4-1-1 Ogawahigashi-cho, Kodaira, Tokyo, Japan; 4 Department of Neurodevelopmental Disorders, Nagoya City University Graduate School of Medical Sciences, 1 Kawasumi, Mizuho-cho, Mizuho-ku, Nagoya, Japan

**Keywords:** Eating disorders, Adolescence, Early adulthood, Parent, Interpersonal psychotherapy, Interpersonal role, Role transition, Active listening

## Abstract

**Background:**

In cases of adolescent and early adulthood eating disorders, despite the importance of the patients’ relationship with their parents, conflict and confusion frequently occur among them. Interpersonal psychotherapy (IPT) is a present-focused psychotherapy that emphasizes the interpersonal context of symptoms. We developed a remote family education and support program exclusively for parents of patients with eating disorders, based on the principle of IPT. The use of IPT is expected to reduce conflicts in the patient-parent relationship. Consequently, parents will be better able to listen to patients, and patients will be better able to express their thoughts and desires. In this study, we describe the protocol for a randomized controlled trial designed to examine the effectiveness of this program in promoting effective communication in their home based on active listening skills of parents of patients with adolescent and early adulthood eating disorders.

**Methods:**

Participants will be parents of patients aged 12–29 years with adolescent and early adulthood eating disorders. Individually randomized, parallel-group trial design will be employed. Seventy participants will be allocated to one of two treatment conditions: (1) remote family education and support program (four, 150 min weekly group sessions) for parents plus treatment-as-usual for patients (consultation by physicians or no treatment), or (2) waiting for the control condition (parents will wait to start the program for 8 weeks) plus treatment-as-usual for patients. The primary outcome measure will be parents’ active listening ability as measured by the Active Listening Attitude Scale at 8 weeks after randomization. Additionally, perception of social support (Social Provision Scale-10 item), loneliness (UCLA Loneliness Scale), mental health status (K6), family function (Family Assessment Device), and parent-evaluated eating disorder symptoms (Anorectic Behavior Observation Scale) will be assessed. Data from the intention-to-treat sample will be analyzed 8 weeks after randomization.

**Discussion:**

This is the first study to evaluate the effectiveness of a family education and support program for parents of patients with adolescent and early adulthood eating disorders based on IPT. If this type of intervention is effective, although indirect, it could be a new support method for this patient population.

*Trial registration:* Clinical Trials. gov ID NCT05840614.

## Abstract (plain language summary)

For patients with adolescent and early adulthood eating disorders, although the relationship with their parents is an important interpersonal dynamic, conflicts and confusion often arise between patients and their parents. On the other hand, parents who live with individuals with eating disorders are frequently involved in interpersonal disputes, leading to a heavy psychological burden and elevated levels of depression and anxiety. It has been found that highly depressed or anxious parents tend to have difficulty listening carefully to their patients. Additionally, parental anxiety often promotes an overprotective response. Interpersonal psychotherapy (IPT) is a present-focused psychotherapy that emphasizes the interpersonal context of symptoms. In IPT, the patient and therapist work within interpersonal therapeutic domains, such as interpersonal role disputes with different expectations and role transitions. We developed a remote family education and support program exclusively for parents of patients with eating disorders based on IPT principles. In the present study, we describe the protocol for a randomized controlled trial designed to examine the effectiveness of this program in promoting effective communication within their homes, focusing on the active listening skills of parents of patients with adolescent and early adulthood eating disorders.

## Background

Eating disorders are serious mental disorders with peak onset in adolescence associated with high mortality, disability, and physical and psychological morbidity [1, 2]. Particularly common issues are interpersonal problems affecting their ability to form healthy and intimate relationships with others during adolescence and early adulthood. Patients with eating disorders have been found to display more interpersonal problems compared with non-clinical samples [3], which contributes to the maintenance of eating disorder symptoms; the degree of interpersonal problems is positively associated with more concerns over eating, shape, and weight, as well as the severity of bulimic behaviors [4].

For patients with adolescent and early adulthood eating disorders, the relationship with their parents is an important interpersonal relationship. However, parents who live with individuals suffering from eating disorders often find themselves entangled in prolonged interpersonal conflicts, placing a significant psychological burden, particularly on mothers [5]. In a systematic review of the expressed emotion (EE) in eating disorders, caregivers frequently exhibit high levels of EE, particularly emotional overinvolvement (EOI) [6]. EE is an index reflecting the familial relationship and is assessed by examining the emotions expressed towards the patient by family members. High EE among caregivers is considered a natural response in situations where caregivers have little information about their patient’s treatment, feel ill-equipped to cope, and experience isolation without adequate support. Although several effective treatments for eating disorders exist [7–9], it remains challenging to provide these for everyone in need. In the context of eating disorders, high EE among caregivers has been associated with increased anxiety and depression [10], heightened caregiver distress [11], and diminished caregiving skills [11]. Although several studies suggest that parents’ EE may not consistently predict treatment outcomes, particularly in adolescents [12–14], numerous others indicate that high EE in caregivers may negatively impact outcomes of ED treatment [15–17]. In addition, many studies suggest that parents of individuals with eating disorders experience elevated levels of depression and anxiety [10, 18–20]. It has been found that highly depressed or anxious parents may struggle to effectively communicate with their children. Among mothers of individuals with eating disorders, there is a significant negative correlation between depression or anxiety and their ability to actively listen as measured by Active Listening Attitude Scale (ALAS) [21]. Moreover, parental anxiety often triggers an overprotective response that manifests as EOI. Parents’ anxiety may lead to behaviors characterized by excessive attention, over-adaptation to the situation, or attempts to conceal negative patient outcomes [10, 22].

Interpersonal psychotherapy (IPT) is a time-limited and affect-, life-event-, and present-focused psychotherapy originally conceptualized for depression [23]. In IPT for depression, the patient and therapist work collaboratively on one (or two) of the following four interpersonal therapeutic foci: (1) lack of intimacy and interpersonal deficits; (2) interpersonal role disputes with different expectations (a conflict, overt or covert, in an important relationship); (3) role transitions; and (4) complicated grief. In addition, family-based IPT (FB-IPT) was developed as a treatment approach for preadolescent depression [24, 25]. FB-IPT focuses on improving parent-patient conflict to decrease preadolescent depression. FB-IPT integrates individual meetings with at least one parent to provide psychoeducation, introduce parenting tips, and set expectations for helping patients maintain daily routines. A prior trial demonstrated that preadolescent patients with depression who underwent FB-IPT were more likely to achieve remission post-treatment than those who underwent child-centered therapy (66% vs. 31%) [25]. In the RCT on preadolescents with loss-of-control-eating, patients in FB-IPT (12 weekly, 45-min session delivered to parent-patient dyads) had a greater reduction in disordered-eating attitudes, as measured by the Eating Disorder Examination adapted for Children at six-months, compared to family-based health education as a control intervention [26].

Considering the characteristics of adolescent and early adulthood patients with eating disorders, their parents are likely to encounter issues related to interpersonal role disputes with different expectations and role transitions within the therapeutic framework of IPT. Additionally, patients with eating disorders have been found to lack confidence in identifying their thoughts and feelings [27, 28]. Consequently, they may struggle to assert their thoughts and desires appropriately and may experience difficulty in expressing themselves to others [3, 4, 29]. As a result, communication between patients and parents in the home may become conflicted and confused due to these features of eating disorders. In such a situation, it is difficult for both patients and parents to clearly recognize their own desires or to guess the other person’s expectations. Additionally, adolescent patients with eating disorders and their parents have difficulty adjusting to the role transition that accompanies physical and mental growth. If parents can listen attentively without experiencing anxiety and communicate with patients in a way that recognizes the role transition associated with adolescent growth, it is expected to lead to improved patient-parent interpersonal relationships and allow patients to cope and challenge themselves with a variety of issues in age-appropriate ways without parents’ overprotection.

In the present study, we developed a family education and support program exclusively for the parents of patients with eating disorders using IPT, focusing on the interpersonal role dispute with differences in expectations and role transitions. It is evident that FB-IPT, targeting both patients and parents, is more effective in treatment. However, treatment resistance and drop out from treatment are common features of eating disorders [30, 31], and initiating and maintaining treatment of eating disorders is extremely difficult. Nevertheless, considering that parent-focused treatment, which addresses only the parents, has been shown to be as effective as family-based treatment (FBT), which involves the entire family, including patients, we believe it is worthwhile to approach only the parents using the IPT concept [32].

In the present study, we designed a pilot randomized controlled trial (RCT) to examine the effectiveness of the remote family education and support program in promoting effective communication within their homes, focusing on the active listening skills of parents of patients with adolescent and early adulthood eating disorders.

## Methods

### Design overview

**Fig. 1:**
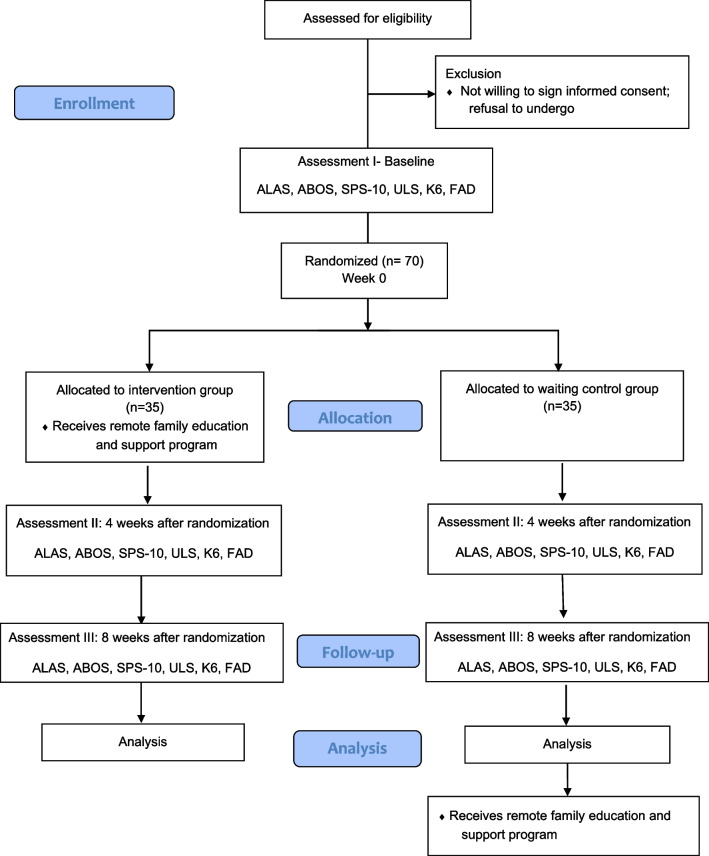
Study flowchart. ALAS, Active Listening Attitude Scale; ABOS, Anorectic Behavior Observation Scale; SPS-10, Social Provision Scale 10 item; ULS, University of California, Los Angeles Loneliness Scale; K6, Kessler Psychological Distress Scale; and FAD, Family Assessment Device

This pilot RCT will be conducted on parents of patients with eating disorders who will be allocated to one of two arms: (1) remote family education and support program in addition to treatment-as-usual for patients and (2) treatment-as-usual for patients only. Treatment-as-usual consists of consultation administered by a physician or no treatment. The primary endpoint is improvement in parent’s active listening ability, as measured by the Active Listening Attitude Scale at 8 weeks (Fig. 1, Table 1).

**Table 1:**
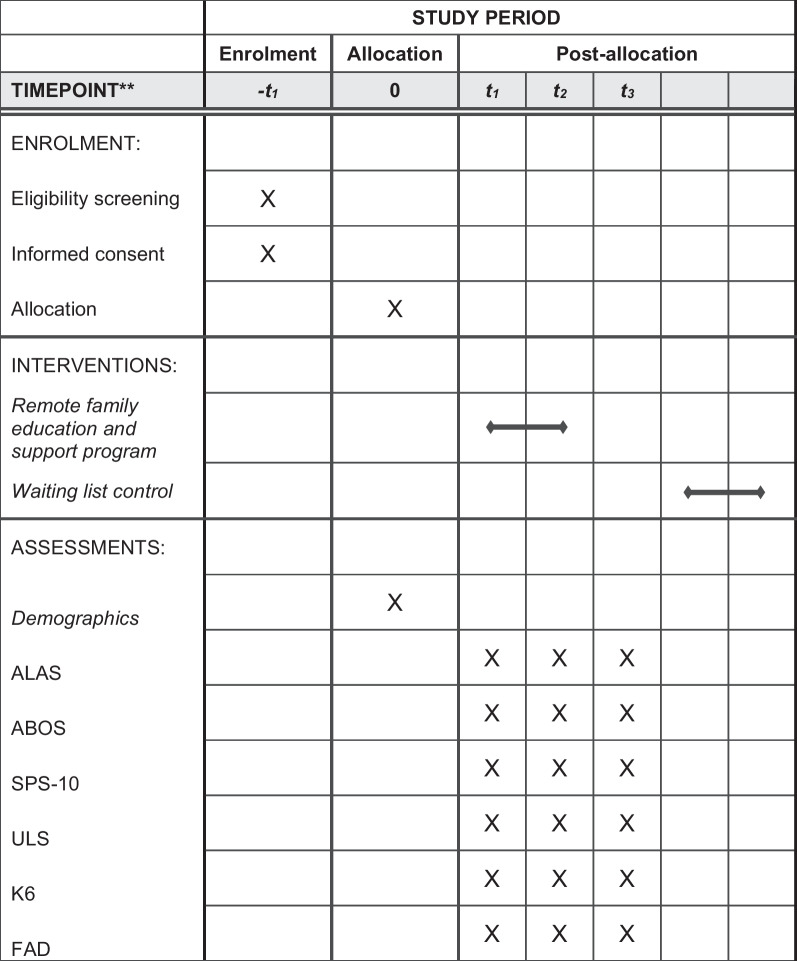
Schedule of enrolment, interventions, and assessments

ALAS, Active Listening Attitude Scale; ABOS, Anorectic Behavior Observation Scale; SPS-10, Social Provision Scale 10 item; ULS, University of California, Los Angeles Loneliness Scale; K6, Kessler Psychological Distress Scale; and FAD, Family Assessment Device

The study protocol was approved by the Ethics Review Committee of Nagoya City University Graduate School of Nursing. All participants will be asked to provide written informed consent after the purpose and procedures of the study have been explained. This study is registered at ClinicalTrials.gov under number NCT05840614. The study was designed in accordance with the SPIRIT statement [33].

### Eligibility criteria

The target population will be parents of patients with adolescent or early adulthood eating disorders. The inclusion criteria will be as follows: (1) parents of patients who have been diagnosed with eating disorders (anorexia nervosa; AN and bulimia nervosa; BN) by a physician or have symptoms of an eating disorder (parents may or may not be biological parents); (2) parent-rated Anorectic Behavior Observation Scale (ABOS) score > 8 points at enrollment; (3) patient age between 12 and 29 years at enrollment; (4) parents who live with patients at the time of enrollment and are expected to live with them during the study period; (5) patients with other psychiatric comorbidities; and (6) parents with other psychiatric comorbidities. Patients may or may not undergo any kind of treatment. If multiple family members (e.g., father and mother) participate in the program, a primary participant will be determined; that person will be the target research participant. The reason for targeting both AN and BN is that family dysfunction due to inadequate communication skills of parents is present in families with both diagnoses and is related to patient symptoms [34, 35]. The reason for targeting the age range of 12–29 years is that even though individuals may be in their late twenties, they often have not completed the developmental tasks of adolescence due to eating disorders and may remain adolescent-minded. Social and economic changes, such as increased access to third-level education and improvements in reproductive health, have led to delays in achieving key milestones of adulthood (e.g., marriage, home ownership), resulting in a growing consensus amongst developmental researchers that adulthood is not achieved until the mid-twenties [36, 37]. Therefore, individuals in their twenties continue to transition into adulthood. Moreover, in the present study, we target parents and patients who live together; thus, conflict within the family may persist, even if they are in their late twenties. The following will be the exclusion criteria: (1) parents who cannot read or write in Japanese; (2) parents who cannot use the Zoom online meeting system; and (3) study investigators and their families. No treatment will be excluded from consideration. This decision is based on the desire to adapt the study results as an effective study for families with eating disorders at all stages of treatment. Even FBT, which has the most empirical support in the treatment of adolescent AN [9], is not effective for all patients, with only 30–50% achieving full remission at the end-of treatment [38, 39].

### Procedure

We will recruit participants using our Japanese-language website and flyers. Participants will sign up via e-mail. We will provide eligible participants with an ID number and ask them to provide informed consent and complete a baseline assessment. After the baseline assessment, participants will be randomized. Assessments will be performed at baseline (Assessment I), before randomization, and at 4 weeks (Assessment II) and 8 weeks (Assessment III) after randomization (Fig. 1). The trial period will be from week 0 to week 4, and the follow-up period from week 4 to week 8. Assessment II will be performed at the week 4 evaluation date ± 5 days, and Assessment III at the week 8 evaluation date ± 5 days.

### Randomisation

Participants will be randomly allocated to one of the two groups in a 1:1 ratio. A computer randomization system will generate the random allocation sequences using minimization and stratify participants according to their ability to engage in active listening (ALAS score cutoff of 36 points).

### Treatment

#### Intervention

The remote family education and support program will include group therapy divided into four sessions (Table 2). Each session will consist of a lecture, followed by role- playing and supportive group therapy, with a duration of approximately 150 min. The groups will comprise approximately six to nine members, and will meet once a week over the course of 4 weeks. Participants will use the Zoom online meeting system in their own homes. The remote family education and support program will consist of IPT elements and family psychoeducation. Family psychoeducation is a method for working with families who are supporting persons with mental illness and has been associated with relapse prevention or improving symptoms [40–42]. Family psychoeducation is more than just providing information. It also focuses on the development of problem-solving, communication, and coping skills. Studies on family psychoeducation for eating disorders have shown that caregivers report an increase in knowledge about eating disorders, an enhancement in their self-efficacy, and a reduction in EE, particularly EOI, as well as psychological and family distress [43, 44]. We will provide participants with information on symptoms of eating disorders with the goal of externalizing the illness. In the first session, we will inform the participants about the symptoms of eating disorders and the mechanism of IPT; in the second session, we will share details about the characteristics of adolescents; and in the third and fourth sessions, we will provide participants with information on effective communication based on IPT. During the third and fourth sessions, we will explain the mechanism of interpersonal role disputes with differences in expectations and the role transition with adolescent growth using an adolescent eating disorder case. Subsequently, participants will role-play using IPT communication skills. Supportive group therapy using problem-solving focuses on members’ strengths and on coping ideas.

**Table 2:** Overview of the remote family education and support program content

	Lecture	Role-play	Support group therapy
	30 min	30 min	90 min
Session 1	Information on the symptoms of eating disorders and the mechanism of IPT	None	
Session 2	Information on the characteristics of adolescents, focusing on adolescent role transitions	None	
Session 3	Information on communication according to IPT. We will explain the mechanism of interpersonal role disputes with differences in expectations using a case	Role-play using IPT communication skills (i) Group members will image how the parent in the case feels in one scene of an eating disorders parent-patient case and share it with group members (ii) In the same scene, group members will image what the parent in the case is expecting from the patient and share it with group members (iii) In the same scene, group members will image how the patient in the case feels and share it with group members (iv) In the same scene, group members will image what the patient in the case is expecting from the parent and share it with group members (v) In the same scene, group members will think about what they would say to this patient and share it with group members (vi) Role play: one of the group members plays the role of the parent	Supportive group therapy using problem-solving focuses on members' strengths and coping ideas (i) Families will be socializing with other families to warm up (ii) Group members will present their own problems or goals (iii) For each problem or goal, group members will discuss and suggest possible solutions based on the person's strengths
Session 3	Information on effective communication according to IPT. We will explain the mechanism of role transition due to adolescent growth using a case	Role-play using IPT communication skills Above, (i) ~ (iv) are the same (v) In the same scene, group members will think about what they would say to this patient, being aware of the role transition that accompanies adolescence, and share it with group members (iv) Role play: one of the group members plays the role of the parent	

IPT, interpersonal psychotherapy

#### Control condition

Participants allocated to the waiting control group will wait to start the remote family education and support program for 8 weeks. There will be no intervention in the control group.

#### Therapist training/supervision and fidelity control

Three therapists will conduct the program. The first author (FK) is a nurse and family therapist certified by the Japan Network of Psychoeducation and Family Support Program [45] and received IPT first class workshop certification. The fourth author (HS) is a nurse and psychologist. The third therapist is a nurse. All therapists have psychiatric nursing experience. The third author (MK) is a certified supervisor authorized by the International Society of Interpersonal Psychotherapy and will supervise this program. To ensure the fidelity of each session, all sessions will be recorded on a computer, and 25% of each condition will be randomly selected, evaluated, and commented on for continuous improvements by an independent researcher.

### Outcome measures for parents

#### Baseline characteristics

The following baseline sociodemographic characteristics will be obtained: (1) age (parent’s and patient’s); (2) patient’s eating disorder duration; (3) patient’s sex; (4) family relationship (father, mother, and other); and (5) whether parents participate in a family self-help group.

### Primary outcome measure for parents

This program aims to improve the confusion and stagnation in patient-parent communication within the home, focusing on enhancing parents’ active listening abilities. Thus, we selected the ALAS score as the primary outcome measure.

#### Active Listening Attitude Scale (ALAS*)*

The ALAS, developed by Mishima et al.[46], will be used to assess the listening attitude. The ALAS comprises 20 items in two subscales: listening attitude (10 items) and listening skills (10 items). Each item is scored from 0 to 3; the higher the score, the better the listening attitude or skill. Cronbach’s α of the ALAS in our previous study was 0.855 [47].

### Secondary outcome measures for parents

#### Anorectic Behavior Observation Scale (ABOS)

The ABOS is a questionnaire used to evaluate patients’ eating behavior based on information provided by their relatives. The ABOS consists of 30 items to be answered ‘yes’ (2 points) or ‘no’ (0 points) if relatives are certain of the information, or ‘?’ (1 point) if relatives are uncertain. The higher the total score, the more pathological the subject’s behavior is considered to be. The ABOS has three domains: (a) eating behavior, concern with weight and food, denial of the problem; (b) bulimic-like behavior; and (c) hyperactivity. The ABOS has a sensitivity of 90.0% and a specificity of 89.6% [48]. The reliability and validity of the Japanese version of the ABOS has been confirmed with Cronbach’s α value of 0.82 [49].

#### Social provisions scale-10 item (SPS-10)

The perception of social support will be evaluated using the shortened version of the Social Provisions Scale (SPS), originally created by Cutrona and Russell [50] and later shortened (SPS-10) by Iapichino et al. [51]. We created a Japanese version of the SPS-10 [52]. The SPS-10 consists of 10 items and retains the following five of the six original SPS subscales: attachment (emotional support), social integration, reassurance of worth, reliable alliance (material support), and guidance. The total SPS-10 score can range from 10 to 40, with a higher score indicating a stronger perceived provision of social support. Cronbach’s α of the original Italian version was 0.809 [51], and that of the Japanese version was 0.89 [52].

#### University of California, Los Angeles loneliness scale (ULS)

To assess loneliness, we will use the Japanese version [53] of the University of California, Los Angeles Loneliness Scale (ULS), originally developed by Russell et al. [54]. The ULS is a 20-item scale, with each item scored from 1 to 4; the higher the score, the stronger the loneliness. Cronbach’s α of the Japanese version was 0.885 [55].

#### Kessler psychological distress scale (K6)

To assess psychological distress, we will use the Japanese version [56] of the Kessler Psychological Distress Scale (K6), originally developed by Kessler et al. [57]. The K6 is a 6-item self-reported scale that was developed to screen for depression and anxiety disorders based on definitions from the Diagnostic and Statistical Manual of Mental Disorders (Fourth Edition), and it analyzes patients’ symptoms over the last 30 days. Moreover, it can be used to quantify nonspecific psychological distress [57]. Items are rated from 0 to 4, and the total score ranges from 0 to 24; the higher the score, the more severe the psychological distress. Two independent studies have analyzed and confirmed the excellent validity of the K6 [57, 58], and the validity of the Japanese version has also been confirmed [56]. Cronbach’s α of the original tool was 0.89 [57].

#### Family Assessment Device (FAD)

To assess family functioning, we will employ the Japanese version of the Family Assessment Device (FAD) [59], developed by Epstein et al. [60]. The FAD is used to evaluate a family system based on the MacMaster model of family functioning, and comprises seven subscales. We will use only the General Functioning subscale. Each item will be scored from 1 to 4; the higher the score, the poorer the family functioning. Cronbach’s α of the General Functioning subscale was 0.92 in the original study [60] and 0.85 in another study [61]. The Family Questionnaire [62], typically used to assess family functioning in eating disorders studies [63, 64], was not chosen for the present study due to the unavailability of a Japanese version.

### Sample size and statistical power

The sample size was calculated based on a power analysis conducted for the ALAS score. Effect sizes were estimated from our previous cohort study [21]. The change in ALAS scores from pre-treatment to post-treatment (8 weeks after randomization) was 7 ± 6.0 (mean ± SD) in the intervention group and 1 ± 6.0 in the waiting control group. With a power of 0.9 to detect a significant difference at P = 0.05 (two-sided), it was calculated that 28 patients would be required for each arm. Thus, allowing for a 20% dropout rate, 35 participants will need to be recruited per group.

### Data analysis plan

Statistical analysis will be performed using IBM SPSS Statistics v.26 for Windows (IBM Corp., Armonk, NY, USA). The initial descriptive statistics will summarize participant characteristics as means, standard deviations, minima, maxima, frequencies, and percentages. All analyses will be based on the intent-to-treat model. We will use analysis of covariance at 8 weeks if there is no missing data at that time. If missing data is observed, we will use the maximum likelihood mixed model, which accounts for missing data, provided that the data are missing at random, conditional on the covariates and the baseline values of the outcome. *P* < 0.05 will be set to test the null hypothesis.

## Discussion

This paper describes the study protocol for an RCT designed to investigate the effectiveness of a remote family education and support program using IPT for parents of patients with adolescent and early adulthood eating disorders. The primary outcome of this study is the active listening ability of parents at 8 weeks after randomization, and secondary outcomes include eating disorder symptoms of patients observed by parents, loneliness, social support, mental health status, and family functioning.

In patients with eating disorders, interpersonal problems are common. In particular, adolescent and early adulthood patients and their parents have difficulty in domestic communication and adjusting to the role transition, because adolescent patients undergo large and rapid changes due to physical and mental growth. The IPT concept (including interpersonal role dispute with different expectations and role transition) is expected to fit well the adolescent and early adulthood patients with an eating disorder and their parents.

The current study has several strengths. First, it is the first attempt to use IPT theory for a parental education and support program for eating disorders. Second, we created a program focusing on the role transition through changes in adolescence and early adulthood. Third, this program is a remote participation program; therefore, participants can join from anywhere in Japan. In Japan, the treatment system for eating disorders is inadequate, and with only five treatment base hospitals in the country. Many patients do not receive adequate treatment because hospitals with specialists in the treatment of eating disorders are far away. Therefore, their parents are also living with anxiety and loneliness. Fourth, we will assess not only the parents’ status but also the eating disorder symptoms of patients, as measured by the ABOS. The ABOS is a useful instrument for eating disorders with high sensitivity and specificity [48]. This trial also has limitations. Patients themselves cannot participate in this program in the current study. Naturally, treatment is best conducted with both patients and their parents according to the FB-IPT, particularly in the treatment of adolescents. Further studies will be designed for both patients and parents to participate in the program.

## Data Availability

The datasets used and/or analyzed in the current study are available from the corresponding author upon reasonable request.

## Abbreviations

ABOS: Anorectic Behavior Observation Scale
ALAS: Active Listening Attitude Scale
EE: Expressed emotion
EOI: Emotional overinvolvement
FAD: Family Assessment Device
FB-IPT: Family-based interpersonal psychotherapy
IPT: Interpersonal psychotherapy
K6: Kessler Psychological Distress Scale
RCT: Randomized controlled trial
SPS-10: Social Provision Scale 10 item
ULS: University of California, Los Angeles Loneliness Scale
